# High yield recombinant thermostable α-amylase production using an improved *Bacillus licheniformis *system

**DOI:** 10.1186/1475-2859-8-58

**Published:** 2009-10-31

**Authors:** Dandan Niu, Zhirui Zuo, Gui-Yang Shi, Zheng-Xiang Wang

**Affiliations:** 1 Center for Bioresource and Bioenergy, School of Biotechnology & Culture and Information Center of Industrial Microorganisms of China Universities, Jiangnan University, Wuxi 214122, PR China; 2 The Key Laboratory of Industrial Biotechnology, Ministry of Education, Wuxi 214122, PR China; 3 Institute for Wine Biotechnology, University of Stellenbosch, Matieland 7602, South Africa

## Abstract

**Background:**

Some strains of *Bacillus licheniformis *have been improved by target-directed screening as well as by classical genetic manipulation and used in commercial thermostable α-amylase and alkaline protease production for over 40 years. Further improvements in production of these enzymes are desirable.

**Results:**

A new strain of *B. licheniformis *CBBD302 carrying a recombinant plasmid pHY-amyL for *Bacillus licheniformis *α-amylase (BLA) production was constructed. The combination of target-directed screening and genetic recombination led to an approximately 26-fold improvement of BLA production and export in *B. licheniformis*. Furthermore, a low-cost fermentation medium containing soybean meal and cottonseed meal for BLA production in shake-flasks and in a 15 liter bioreactor was developed and a BLA concentration of up to 17.6 mg per ml growth medium was attained.

**Conclusion:**

This production level of BLA by *B. licheniformis *CBBD302(pHY-amyL) is amongst the highest levels in Gram-positive bacteria reported so far.

## Background

In any industrial biotechnological process, achieving high productivity is an essential factor for commercial success. The maximum specific productivities of a production strain are usually independent of process parameters and determined by the genetic and physiological properties of the organism. Bacterial extracellular enzymes are an important class of industrial enzymes constituting approximately 20% of the enzyme market [1,2]. To obtain a high yield in bacterial extracellular enzyme production, the following genetic and physiological properties of the strain are important: a) the metabolic flux for amino acids synthesis and ATP regeneration, b) the cell growth rate and cell density in an inexpensive medium, c) mainly vegetative growth by spore-forming strains, d) secretion capacity for extracellular enzymes, e) long-term preservation in an active form in broth, and f) a high expression level of the specific gene encoding a bacterial extracellular enzyme [3].

Genetic improvement of bacterial extracellular enzyme production is achieved by applying a range of strategies based on molecular cloning tools. These include: 1) enhancement of expression level through amplification of gene copy number [4], codon usage optimization [5], or strong promoters being used to boost gene transcription [6]; 2) enhancement of secretion by modulation of signal peptides [7,8], fusion to heterologous signal peptides for efficient targeting to the translocase [9], increasing the copy number of signal peptidase genes [10,11], or deregulation and/or co-expression of chaperon encoding genes to make efficient protein folding [12]; 3) improvement of productivity through re-designing the capacity of the secretion machinery by targeted deletion of genes encoding non-beneficial extracellular enzymes [13], or genome reduction [14]; and 4) improvement of the productivity by preventing degradation of extracellular enzymes using protease deficient strains [15].

In general, a specific strain should possess a definite maximum capacity for synthesis and secretion of extracellular enzymes [4]. Overproduction of secreted proteins sometimes severely affects the secretory system of an organism [16] and eventually results in a secretion stress response that may limit secretion [17,18]. Moreover, it is almost impossible at the present stage to enhance the maximum synthesis and secretion capacity by site-directed gene disruption and expression in a specific strain. Alternatively, data from genome shuffling and genome size reduction studies [14] strongly suggest that a natural strain should exist with a maximum capacity for synthesis and secretion of extracellular enzymes as a result of accumulation of mutations and shuffling of the genomes.

In present work, the combination of target-directed screening and genetic recombination yielded a novel *B. licheniformis *strain that produced up to 17.6 mg *B. licheniformis *α-amylase per ml growth medium.

## Results and discussion

### Rationale for the selection approach of the *B. licheniformis *strain and property identification

Enzyme synthesis and export is an energy-dependent event [3]. In order to select a suitable host cell for BLA overproduction, following genetic and physiological criteria were applied: 1) the ability of the strain to sporulate should be poor in order to extend the duration of BLA production; 2) the strain should form little or no lichenysin in order to reduce consumption of ATP and the amino acid pool; 3) the strain should not clump during cultivation to maintain efficiency in a production bioreactor; 4) the strain should grow well on either low-cost fermentation medium containing a high substrate concentration appropriate for an industrial process; 5) the strain should contain no native plasmids but be sensitive to kanamycin or tetracyclin to facilitate further genetic manipulation and 6) the strain should be amenable to transformation to enable genetic modification.

### *B. licheniformis *strain selection and biological property identification

**Figure 1:**
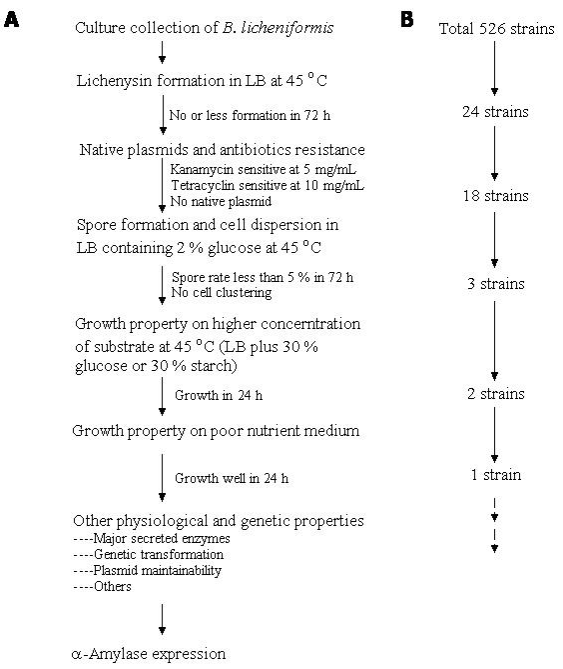
***B. licheniformis *strain selection strategy and selection**. A: selection routine; B: selection results.

**Figure 2:**
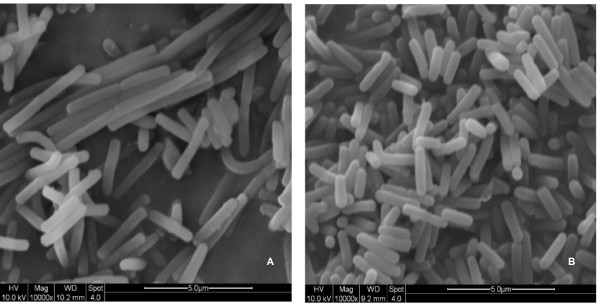
**The scanning electron micrograph of *B. licheniformis***. 10^8 ^cells were inoculated in a 250 ml flask with 30 ml of LB medium and cultivated for 10-12 h at 45°C and 220 rpm. A drop of culture was collected and analyzed by scanning electron microscope. A: *B. licheniformis *CICIM B30306; B: *B. licheniformis *CBB0302.

Following a selection procedure described above, based on these criteria, a candidate strain designated as CBB0302 was selected out of a total of 526 *B. licheniformis *isolates (Figure 1). The strain produced catalase, amylase and protease, utilized citrate, propionate and nitrate, grew in 7% NaCl, and at 50°C but not at 60°C, typical of *B. licheniformis *strains. Notably, fewer than 0.5% of the cells in culture formed spores and no cell clumping was found after 72 h cultivation. Furthermore the strain did not produce lichenysin-like pigments and harboured no native plasmids. The cell shape of the strain CBB0302 was identical to that of *B. licheniformis *CICIM B30306, an industrial BLA-producing strain, when both strains were cultivated in LB medium for 10-12 h at 45°C and 220 rpm but the average cell volume was 30-40% less (Figure 2).

### Increased transformation efficiency by genetic modification

The transformation efficiency of the natural *B. licheniformis *cells is poor and strains routinely need long periods with difficulty to finally obtain a desired transformant [[19]; our own unpublished results]. This is mainly due to the existence of two type I restriction modification systems (RMS) in *B. licheniformis *[20,21]. Single as well as double knock-outs of the RMS resulted in strains being readily transformable with plasmids isolated from *Bacilli*. Introduction of shuttle plasmids isolated from *Escherichia coli *is routinely possible when the double mutant *B. licheniformis *MW3 (Δ*hsdR1*, Δ*hsdR2*) was used in transformation experiments [19].

A strain CBBD302 was developed by deletion of a type I RMS locus in strain CBB0302 by using homolog-mediated recombination according to the method described by Waschkau *et al *[19]. The growth and secretion of extracellular enzymes by this strain were unaffected by the deletion (data not shown). The transformation efficiency with a shuttle plasmid pHY-300PLK isolated from *E. coli *was significantly improved and 42 cfu/μg DNA in strain CBBD302 but only 5 cfu/μg DNA in strain CBB0302 were attained.

### Increased recombinant BLA production and secretion by using *B. licheniformis *CBBD302 as host

**Figure 3:**
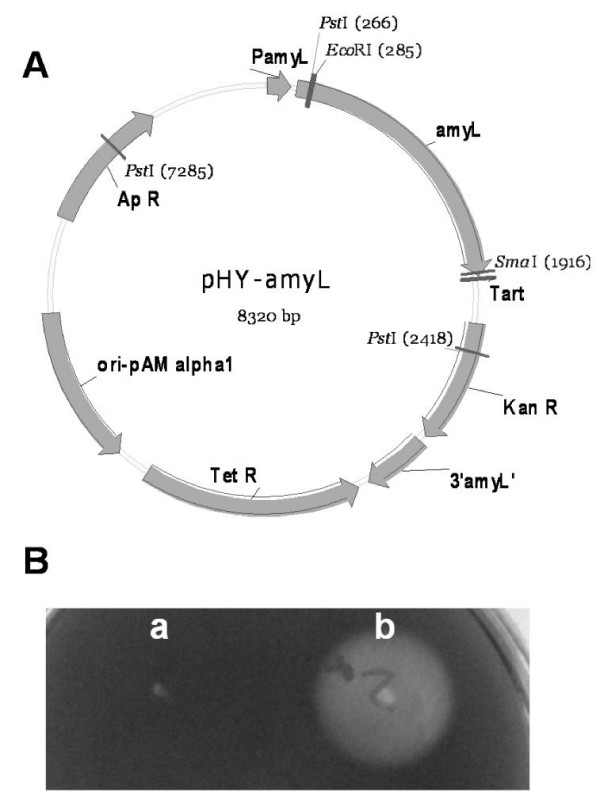
**Development and identification of pHY-amyL**. A: The physical map of pHY-amyL. B: α-Amylase was expressed by *E. coli *carrying pHY-amyL, a: *E. coli*(pHY-WZX); b: *E. coli*(pHY-amyL).

**Figure 4:**
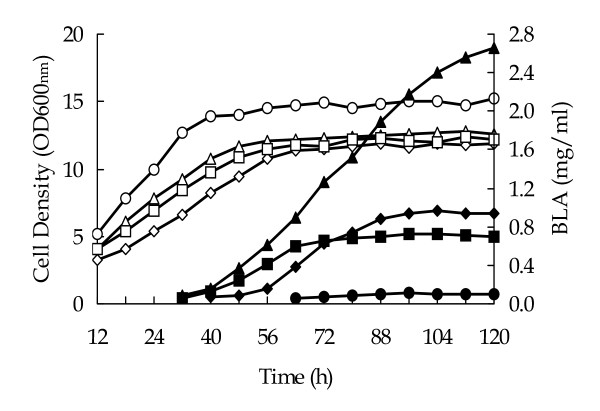
**BLA production by *B. licheniformis *CBBD302(pHY-amyL)**. *B. licheniformis *CBBD302 (circles), *B. licheniformis *CBBD302(pHY-amyL) (triangles), *B. licheniformis *B30306 (squares) and *B. licheniformis *B0204 (diamonds) were cultivated in LB medium supplemented with 40 g/l lactose for up to 120 h at 42°C and 220 rpm. Open symbols for cell growth and closed symbols for BLA production.

In order to test BLA production in *B. licheniformis *CBBD302, a recombinant plasmid pHY-amyL was constructed. A 1.6 kb fragment containing *B. licheniformis *B0204 *amyL *coding for the mature BLA peptide was recovered from pET28a-amyL_NEW _by PCR, inserted into the *Eco*RI and *Sma*I sites of pHY-WZX and functionally tested in *E. coli *(Figure 3), yielded hybrid plasmid pHY-amyL. Subsequently, pHY-amyL was transferred into *B. licheniformis *CBBD302, yielding *B. licheniformis *CBBD302 (pHY-amyL). The BLA production was carried out in LB supplemented with 40 g/l lactose in shake-flasks (Figure 4). *B. licheniformis *CBBD302, *B. licheniformis *B30306, *B. licheniformis *B0204 and *B. licheniformis *CBBD302 (pHY-amyL) produced 0.1, 0.7, 0.9 and 2.6 mg BLA per ml growth medium, respectively. Strain CBBD302 carrying pHY-amyL produced a 26-fold improvement in BLA production compared to the parent strain CBBD302 and about three times compared to the *B. licheniformis *B0204. Although the growth rate was slower than its parent CBBD302, strain CBBD302 (pHY-amyL) grew significantly faster than industrial strains B0204 and B30306 did (Figure 4).

Bacterial extracellular enzyme production is a complex process, in which the efficiencies of transcription and translation of the enzyme-encoding genes as well as protein translocation define the enzyme concentration in the growth medium and are under control of the bacterial host. A *B. licheniformis *strain has been found to have a specific capacity maximum for protein synthesis and secretion by the introduction of different copy numbers of *amyL *in *B. licheniformis *B0204 [3]. Evidently the capacity could be improved since *B. licheniformis *CBB0302 with physiological properties of less than 0.5% sporulation, no production of lichenysin-like pigments, reduced nutrient requirement for growth, smaller cell volume, identical cell shape and no cell clumping during propagation gave an increased BLA yield.

### Optimization of fermentation medium

**Figure 5:**
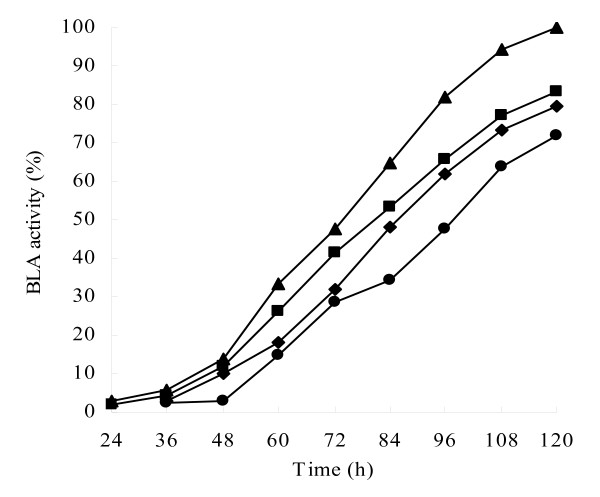
**The time-course for BLA production in *B. licheniformis *CBBD302 (pHY-amyL) on different carbon sources**. Symbols: triangle, lactose; square, glucose; diamond, starch; circle, corn cob hydrolysate. The highest activity of BLA on lactose is designated as 100% activity.

α-Amylase production is subjected to catabolite repression by glucose and other sugars, similar to most inducible enzymes. Therefore, the use of glucose in the production of α-amylase in certain cases is problematic [22,23]. A number of other substrates such as lactose [24] have also been used for the production of α-amylase. The effect of the addition of different carbon sources to the fermentation medium on BLA production was investigated and the results are summarized in Figure 5. BLA secretion by strain CBBD302 carrying pHY-amyL varied between carbon sources and the lactose gave the highest BLA concentration and glucose, starch and corn cob hydrolysate supported a 70~80% BLA production level compared to lactose (Figure 5). These results indicate that many various carbon sources can be used for BLA production and BLA synthesis in CBBD302 is not subject to catabolite repression.

Yeast extract alone or in conjunction with other nitrogen sources such as bactopeptone and ammonium sulfate has been used for the production of α-amylase from *Bacillus *sp. [25]. In this study, the effect of various nitrogen sources in the fermentation medium on BLA production was investigated. When soybean meal, fish meal or (NH_4_)_2_SO_4 _was added as nitrogen source for BLA production to the fermentation medium, lower respective BLA production levels of 95.8, 28.6 and 25.3% were obtained relative to cottonseed meal. Much higher BLA production levels were obtained with cottonseed meal and/or soybean meal supplemented 0.01 mol/l ammonium sulphate as nitrogen source (respectively 132 and 140% of cottonseed level).

**Figure 6:**
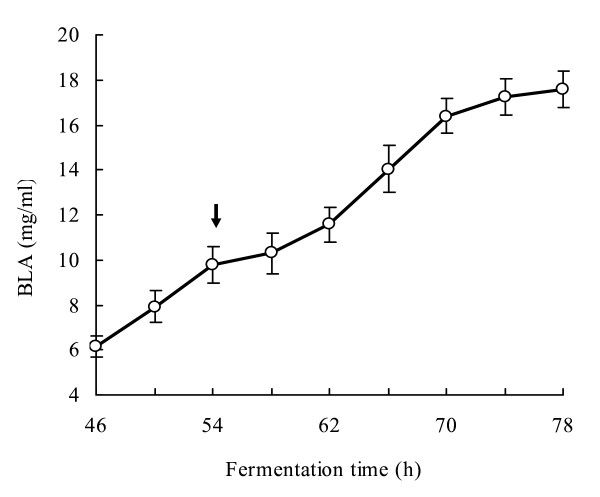
**A time-course of BLA production with *B. licheniformis *CBBD302(pHY-amyL) in 15 l fermentor**. Arrow indicates one more 40 g/L lactose was fed at that point during the fermentation.

The maximum production of BLA by *B. licheniformis *CBBD302(pHY-amyL) of 17.6 mg/ml was obtained when the strain was cultivated in the fermentation medium consisting of 40 g/l lactose, 25 g/l soybean meal, 20 g/l cottonseed meal, 30 g/l corn-steep liquor and 0.01 mol/l ammonium sulfate in a pH-controlled 15 l bioreactor fermentations (Figure 6). The BLA production was boosted by the 40 g/l lactose when fed at the 54^th ^hour.

## Conclusion

The combination of target-directed screening and genetic recombination led to an overall 26-fold improvement of BLA production and export in *B. licheniformis*. In a low-cost fermentation medium containing 40 g/l lactose, 25 g/l soybean meal, 20 g/l cottonseed meal, 30 g/l corn-steep liquor and 0.01 mol/l ammonium sulphate and a 15 l bioreactor up to 17.6 mg BLA per ml growth medium was produced. This production level of BLA by *B. licheniformis *CBBD302(pHY-amyL) is amongst the highest levels in Gram-positive bacteria reported so far.

## Methods

### Plasmids and strains

Plasmid pHY300PLK [26] was used to determine transformation efficiency and plasmid pHY-WZX [27] was used as an expression vector in *B. licheniformis*. *E. coli *XL1 was used as a host cell for functional identification of the recombinant plasmid. Molecular biology methods were described previously [28]. A 1.6 kb fragment containing *amyL *coding for the mature peptide as well as its 180 bp downstream sequence was amplified with primers F2-*Eco*RI (5'-CG**GAATTC**CTTAATGGGACGCTGATGC-3') and R1-*Sma*I (5'-TA**CCCGGG**TACATCAGATAACGTTGCC-3') using pET28a-amyL_NEW _[29] as template. The amplified product was purified and digested with *Eco*RI and *Sma*I and subsequently cloned into the same sites of pHY-WZX to yield recombinant pHY-amyL. *B. licheniformis *isolates as well as B0204 and B030306 were purchased from CICIM-CU . CBBD302 was developed from a native *B. licheniformis *isolate CBB0302, in which the restriction modification system locus was deleted according to the method described by Waschkau *et al *[19]. *B. licheniformis *CBBD302(pHY-amyL) was CBBD302 harboring pHY-amyL by using electroporation [30].

### Strain screening and biological property identification

*B. licheniformis *strains were recovered from a culture collection held at -70°C and single colonies were picked onto LB plate and cultivated at 45°C for up to 72 h. Colonies with no or reduced lichenysin formation were picked onto LB plate supplemented with 5 μg/ml kanamycin or 10 μg/ml tetracycline and cultivated for 48 h. Colonies that failed to grow were examined for the existence of the native plasmids by plasmid extraction and agarose electrophoresis as described previously [28]. Strains carrying no native plasmids and sensitive to kanamycin and tetracycline were cultivated in LB containing 2% glucose at 45°C for up to 72 h and their degree of sporulation and cells dispersion were checked by light microscopy. Strains with sporaulation rate less than 5% after 72 h and with no cell clumping were inoculated onto LB supplemented 30% glucose or 30% starch and cultivated at 45°C for 24 h. The rapidly growing strains were selected and cultivated at 45°C and 24 h on a nutrient limited medium consisting of 0.02% peptone, 0.01% yeast extract, 1% NaCl and 0.01% glucose. Those strains that grew well on the nutrient limited medium were examined for their biological properties including the major secreted enzymes, genetic transformation efficiency as well as plasmid stability as described by Zhuge & Wang [31]. The cells were examined with a Quanta-200 scanning electron microscope (FEI, Netherland).

### Cultivation

*E. coli *XL1 was cultivated at 37°C in LB medium. As required, 100 μg/ml ampicillin and/or 25 μg/ml kanamycin were added to the medium. *B. licheniformis *B0204, B030306 and CBBD302 were cultivated at 42°C in LB medium. For the shake-flask fermentation evaluation, *B. licheniformis *strains were grown in 500 ml Erlenmeyer flasks containing 50 ml LB supplemented with 40 g/l lactose at 42°C and 220 rpm. For optimization studies the fermentation medium consisted of 30 g/l corn-steep liquor, 30 g/l nitrogen source (cottonseed meal, soybean meal and fish meal, and former two combination with 0.01 mol/l (NH_4_)_2_SO_4 _and 40 g/l) carbon source (lactose, glucose, starch or corn cob hydrolysate) at pH 6.0 was used. For bioreactor cultivation studies, a Biostat (B. Braun, Melsungen, Germany) with a 15 l working volume was used. The bioreactor was inoculated with 5% (v/v) broth and cultivated at 42°C with a controlled pH of 6.0.

### Analytical procedures

For shake flask cultivation, samples for cell density and BLA activity were taken at regular intervals. The optical density (OD_600 nm_) was measured in triplicate with an Ultrospec 3100 pro spectrophotometer (Amersham Pharmacia, UK). SDS-PAGE was performed using a Mini Protean 3 apparatus (Bio-Rad, USA). Proteins were stained by Coomassie Brilliant Blue G250. Directly after sampling, BLA activity was measured spectrophotometrically (Ultrospec 3100 pro, Amersham Pharmacia, UK) as described previously [32]. One unit was defined as the amount of enzyme that hydrolyzes 1 mg water soluble corn starch per minute at 70°C and pH 6.0. The amount of BLA protein (g/ml) in the medium was calculated using BLA specific activity parameter of 1 mg BLA is equal to 996 U.

## Competing interests

The authors declare that they have no competing interests.

## Authors' contributions

DN carried out the molecular genetic studies. ZZ participated in the design of the study and performed the statistical analysis. G-YS participated fermentation experiments. Z-XW conceived of the study and participated in its design and coordination. All authors read and approved the final manuscript.
